# Multi-target litholytic strategy toward calcium oxalate stone therapy: a fundamental study

**DOI:** 10.3389/fphar.2026.1779759

**Published:** 2026-05-13

**Authors:** Shasha Xia, Kaiwen Shen, Hong Yang, Yaoke Li, Weijian Mu, Jun Shen

**Affiliations:** 1 Department of Urology, The Affiliated Hospital of Guizhou Medical University, Guiyang, Guizhou, China; 2 College of Clinical Medical, Guizhou Medical University, Guiyang, Guizhou, China; 3 Department of Urology, Guizhou Hospital of The First Affiliated Hospital, Sun Yat-Sen University, Gui’an New Area, Guizhou, China

**Keywords:** calcium oxalate stone, compound litholytic solution, multi-target therapy, preclinical study, synergistic effect

## Abstract

**Introduction:**

Kidney stones, particularly calcium oxalate stones, present a significant global health challenge due to their high recurrence rate and limited non-surgical treatments. This study aims to develop a novel, efficient, and safe multi-target preparation to address post-surgical residual stones and recurrence.

**Methods:**

Targeted drugs were screened against calcium oxalate stone components—crystals, matrix proteins, and microorganisms—using *in vitro* litholysis. Drug combination efficacy and safety were evaluated via further litholysis and CCK-8 assays. The optimal solution, the Multi-Target Compound Litholytic Solution (MTCLS), was compared with Normal Saline and citrate (TCD) using *in vitro* litholysis rate and micro-CT scans to assess stone volume, surface area, and CT value. Safety was evaluated using CCK-8 assays on human urothelial cells and in SD rat models through blood tests, histopathology, bladder mast cell counts, and immunohistochemistry for Uroplakin III, E-Cadherin, TNF-α, IL-1β, and NLRP3.

**Results:**

The drug screening revealed that both sodium polyphosphate and citric acid monohydrate effectively dissolved calcium oxalate stones, with sodium polyphosphate proving more effective. Ammonium sulfate and trypsin effectively targeted the matrix proteins in these stones, but ammonium sulfate, despite being more effective, was excluded due to high cytotoxicity. Lysozyme and piperacillin-tazobactam sodium were effective against microorganisms in the stones; however, piperacillin-tazobactam sodium was excluded for its instability in the compound solution, while lysozyme was chosen for its stability. Following a comparative analysis of various compatibility schemes, the final components of MTCLS were determined to be sodium polyphosphate, citric acid monohydrate, sodium carbonate, potassium carbonate, trypsin, and lysozyme. Compared to TCD, MTCLS exhibited a slightly higher cell survival rate. No significant differences were observed in liver and kidney function tests, histopathological examinations of gastric and bladder tissues (H&E staining), bladder mast cell counts, or immunohistochemical analyses of bladder Uroplakin III, E-Cadherin, TNF-α, IL-1 Beta, and NLRP3. These findings indicate that MTCLS did not cause significant liver or kidney damage, nor did it induce bladder inflammation or compromise mucosal integrity.

**Conclusion:**

MTCLS exhibited greater dissolution efficacy for calcium oxalate stones compared with citrate *in vitro* and showed acceptable short-term biosafety in a 7-day animal study, suggesting that MTCLS is a promising multi-target candidate and a potential alternative to citrate.

## 1 Introduction

Kidney stone disease is one of the most common diseases of the urinary system (Alshareef et al., 2025). Calcium oxalate stones are the most common type of kidney stones worldwide, accounting for approximately 60%–80% of all cases (Tang et al., 2021; Zhang et al., 2023; Wang Y. et al., 2024). Globally, the incidence of kidney stones is on the rise (Bargagli et al., 2025), and the recurrence rate is alarmingly high, with up to 50% of patients experiencing a recurrence within 5 years of the initial episode (Shpitzer et al., 2025). This condition can lead to severe waist pain, urinary obstruction, hydronephrosis, and urinary tract infections (Bargagli et al., 2025). Additionally, patients with kidney stones have been shown to be at significantly increased risk of progressing to end-stage renal disease (Alshareef et al., 2025). Current clinical treatments primarily focus on stone removal, employing various surgical techniques such as extracorporeal shock wave lithotripsy, ureteroscopic lithotripsy and stone extraction, percutaneous nephrolithotomy, and renal or ureteral incision for stone removal (Xia et al., 2022; Liu et al., 2023). While these methods have achieved varying levels of success and continue to be refined, effectively addressing the high recurrence rate remains a significant challenge.

Given the high recurrence rate of kidney stones, secondary preventive measures are frequently implemented in clinical practice (Šálek et al., 2022). Patients are typically advised to increase fluid intake, maintain appropriate calcium and potassium levels, limit sodium and oxalate intake, and reduce animal protein consumption (Dawson and Tomson, 2012; Pearle et al., 2014; Trombetta et al., 2021). These measures help lower the supersaturation of stone-forming components in urine and elevate the metastable state of urine—where supersaturation occurs without precipitation—thereby reducing the risk of stone formation (Dawson and Tomson, 2012; Pearle et al., 2014). Besides dietary changes, common treatments include thiazide diuretics (Tunnicliffe et al., 2024) and citrate therapy (Phillips et al., 2015; Ruiz-Agudo et al., 2017; Robinson et al., 2020). Citrate forms soluble complexes with calcium, inhibits crystal nucleation, growth, and aggregation, and stabilizes multinuclear complexes and amorphous CaOx phases (Ruiz-Agudo et al., 2017; Siener and Hesse, 2021). It is currently the gold standard for stone treatment (Robinson et al., 2020). However, clinical observations indicate that even with citrate therapy, the recurrence rate of stones remains relatively high, particularly in patients with low baseline citrate levels (Phillips et al., 2015). In recent years, extended-release and liquid potassium citrate formulations have been increasingly used in clinical practice to improve patient adherence and tolerability (Hershenhouse et al., 2025; Li et al., 2025). Studies have shown that extended-release formulations reduce gastrointestinal irritation while maintaining stable urinary citrate excretion (Dai et al., 2023; Li et al., 2025). Additionally, flavored liquid formulations (studied in healthy non-stone-forming adults) have been reported to significantly improve palatability and patient acceptance (Hershenhouse et al., 2025); however, their gastrointestinal side effects and long-term adherence require further investigation in stone-forming populations. Long-term follow-up data indicate that potassium citrate therapy has a favorable metabolic safety profile and effectively reduces urinary supersaturation of uric acid (Crivelli et al., 2026). Nevertheless, these newer formulations still face challenges such as higher costs (Sui et al., 2025), an actual adherence rate of only approximately 64% (Crivelli et al., 2026), and the persistence of gastrointestinal intolerance or risk of hyperkalemia in some patients (Crivelli et al., 2026). At present, non-surgical treatment and preventive options for CaOx kidney stones remain extremely limited.

Litholysis therapy for non-surgical kidney stone treatment has garnered significant interest among urological professionals. Research on litholytic drugs for CaOx stones has primarily focused on ethylenediaminetetraacetic acid (Zhou et al., 2014), hexametaphosphate (Robinson et al., 2020), Niaoshitong pills, potassium sodium hydrogen citrate, and various plant extracts, including Garcinia cambogia extract (Fan et al., 2020), Lysimachia christinae, Phragmites communis, and Polygonum cuspidatum water extractss (Zhang and Ding, 2016). Unfortunately, many of these studies were discontinued due to issues like low dissolution efficiency or notable toxic side effects. As a result, the potential of litholytic drugs for treating CaOx kidney stones remains underexplored. The urgent need now is to develop highly effective and safe targeted litholytic drugs to overcome the current clinical treatment limitations.

CaOx stones consist mainly of crystalline and non-crystalline matrix components. The crystalline portion primarily includes calcium oxalate monohydrate (COM) and calcium oxalate dihydrate (COD) (Chen et al., 2023; Maruyama et al., 2023), which share the chemical formula CaC_2_O_4_. These compounds have a stable crystal structure with a well-defined lattice arrangement (Polat and Eral, 2021) and low solubility in water, posing significant challenges for litholytic therapy. The non-crystalline matrix is predominantly composed of proteins (Negri and Spivacow, 2023), with over 1,000 types of stone matrix proteins (SMP) identified. These proteins are categorized based on charge into strongly anionic, strongly cationic, weakly anionic, and weakly cationic groups (Wesson et al., 2022). Notably, calgranulin B (S100A9) and calgranulin A (S100A8) from the S100 protein family are the most abundant (Negri and Spivacow, 2023). Other matrix components include haptoglobin, osteopontin, uromodulin, vitamin K-dependent protein Z, and histones (Negri and Spivacow, 2023). The primary function of SMP molecules is protein binding (Wesson, 2025), which may facilitate stone formation. Additionally, CaOx stones are often linked to urinary tract infections (Chen and Ren, 2022), with microorganisms like *Escherichia coli*, *Enterococcus*, and *Staphylococcus* present in the stone matrix (Shah et al., 2020; Zhao and You, 2021). Genera such as *E. coli* and *Klebsiella* are noted for their strong biofilm activity (Al-Bayati and Samarasinghe, 2022). Given the complexity of CaOx stone components, we propose that a compound preparation targeting these three main components could serve as an effective dissolving agent for CaOx kidney stones.

Previous studies have demonstrated that the formation of CaOx stones is positively correlated with the urinary excretion of calcium, oxalate, phosphorus, and sodium, while it is negatively correlated with the urinary excretion of urine volume, citrate, potassium, and magnesium (Ferraro et al., 2024). Additionally, phosphate exhibits a certain dissolution effect on CaOx stones (Robinson et al., 2020). Notably, the S100 protein family is known to be soluble in a 100% saturated ammonium sulfate solution under neutral conditions, a property documented since its early description (Moore, 1965) and recognized as a defining characteristic of this protein family in subsequent studies (Cerón et al., 2023). Given the complex composition of CaOx stones, this study proposes a multi-target stone-dissolution strategy. This involves developing a compound preparation that simultaneously targets stone crystals, matrix proteins, and microorganisms. The specific plan includes: for stone crystals, using reagents such as citric acid, magnesium citrate, potassium permanganate, and sodium polyphosphate; for matrix proteins, employing trypsin and ammonium sulfate; and for microorganisms, utilizing drugs like lysozyme, furazolidone, levofloxacin, cefixime, and piperacillin/tazobactam sodium. The rationale for selecting each component is summarized in Table 1. Initially, effective targeted reagents for each stone component were identified, followed by diversified compatibility testing, leading to the development of a Multi-Target Compound Litholytic Solution (MTCLS) characterized by high efficiency and low toxicity. Subsequently, its effectiveness and safety were compared with the current gold standard, citrate.

**TABLE 1:** Rationale for the selection of components in MTCLS.

Component	Intended target/Role	Supporting evidence	Key references
Citric acid monohydrate	Chelation of Ca^2+^; crystal dissolution	Forms soluble complexes with calcium ions	Shanthil et al. (2020)
Sodium polyphosphate	Chelation of Ca^2+^; induction of lattice strain	Can embed into the crystal surface and irreversibly inhibit crystal growth via lattice strain; Chelation of Ca^2+^	Deshwal et al. (2024); Kim et al. (2025)
Trypsin	Degradation of matrix proteins	Specifically cleaves arginine- and lysine-rich proteins (e.g., S100A8, S100A9, osteopontin)	Stephan and Nolan (2016); Neukam et al. (2024)
Ammonium sulfate	Disruption of matrix proteins	Dissolves matrix proteins via the salting-in effect; S100 protein family is soluble in 100% saturated ammonium sulfate	Moore (1965); Cerón et al. (2023)
Lysozyme	Antimicrobial activity; disruption of bacterial cell wall integrity	Hydrolyzes the β-1,4 glycosidic bonds in peptidoglycan, leading to cell wall rupture and bacterial lysis	Ragland and Criss (2017); Yang and Yan (2025)
Piperacillin/tazobactam	Antimicrobial activity; disruption of bacterial biofilm	Broad-spectrum β-lactam combination; active against *E. coli* (including ESBL-producing strains)	Mancini et al. (2025)

## 2 Materials and methods

### 2.1 Stone samples

Postoperative kidney stones that would otherwise have been discarded were collected from patients at the Affiliated Hospital of Guizhou Medical University; all specimens had been removed by percutaneous nephrolithotomy. This study was approved by the Ethics Committee of the Affiliated Hospital of Guizhou Medical University and was conducted in accordance with the Declaration of Helsinki.

Stone composition was analyzed by infrared spectroscopy (Han et al., 2025). Stone samples were first washed with normal saline and then dried at 37 °C. One milligram of each dried sample was mixed with potassium bromide at a 1:200 ratio, placed in an agate mortar, and ground thoroughly and unidirectionally with a pestle. The resulting powder was pressed for 1 min at 20 MPa in a tablet press to produce a thin slice 0.3–0.5 mm thick. The slice was immediately analyzed in an infrared spectrometer (Beijing Rely Analysis Instrument Co., Ltd., China). An automated infrared spectroscopy analysis system (LIIR-20) identified stone types by their characteristic spectral peaks, and calcium oxalate stones were selected. All evaluations were performed by professionals, and the results were double-checked for accuracy.

### 2.2 Animals

In this study, we used 36 adult female SD rats (250 ± 20 g; provided by Beijing Huafukang Biotechnology Co., Ltd., Clean grade, License No.: SYXK (Gui) 2023-0002). All rats were housed in the SPF-level laboratory of the Translational Medical Research Center of Guizhou Medical University, where room temperature was maintained at 20 °C–27 °C and humidity at 50%–70%. Rats had free access to food and water, and a 12-h light/12-h dark cycle was adopted. After 1 week of adaptive feeding, the 36 rats were randomly divided into two primary groups by a random number table according to differences in administration routes. Each primary group was then randomly split into three subgroups based on the intervention: normal saline control (NS), trisodium citrate dihydrate control (TCD), and Multi-Target Compound Litholytic Solution (MTCLS), with six rats per subgroup housed separately. The Research Ethics Review Committee of Guizhou Medical University approved the animal protocol (Approval No. 2403688). Experimental procedures complied with the International Veterinary Editors’ Consensus Author Guidelines on Animal Ethics and Welfare and with applicable local and national regulations.

### 2.3 Cell culture

SV-HUC-1 cells were obtained from Shanghai Institutes of Biological Sciences (Shanghai, China). Cells were cultured in F-12K medium (Procell system, PM150910, China) supplemented with 10% fetal bovine serum (FBS, Procell system, 164210, China), 100 U/mL penicillin, and 100 μg/mL streptomycin (Servicebio, G4003-100ML, China) (Liu et al., 2024; Wang F. et al., 2024). All cells were incubated at 37 °C in an atmosphere containing 5% CO_2_.

### 2.4 *In vitro* stone dissolution assay

This study utilized an *in vitro* simulation system to assess the dissolution efficacy of drug components on calcium oxalate (CaOx) stones. The detailed protocol is outlined below:

CaOx stone samples of similar size and weight were immersed in physiological saline for 24 h to ensure full hydration. Afterward, surface moisture was removed using absorbent paper, and the stones were precisely weighed with an electronic balance (FA series, Shunyu Hengping Scientific Instrument, Shanghai, China). Each stone was then placed in a borosilicate glass test tube (20 × 100 mm) and submerged in 5 mL of a drug solution at predetermined concentrations (Robinson et al., 2020). The incubation occurred in a 37 °C water bath under static conditions to simplify variables, with intermittent agitation of the glass test tubes to simulate physiological movement. The drug solution was refreshed twice daily, at 08:00 and 20:00 respectively. At 20:00 each day, the stones were removed, surface-dried, and reweighed. The experiment lasted for 7 days with triplicate samples per group (n = 3). The dissolution rate (%) was calculated using the formula: [(Initial Mass−Day 7 Mass)/Initial Mass] × 100%. A comprehensive evaluation, integrating quantitative analysis (dissolution rate) and qualitative observations (structural integrity degradation, fragmentation state), was conducted to assess the *in vitro* litholytic efficacy of each test compound. Components demonstrating superior dissolution efficacy were identified.

### 2.5 Cell viability assay

The viability of SV-HUC-1 cells was assessed using the Cell Counting Kit-8 (CCK-8, Solarbio CA1210, China) to evaluate the biosafety profile of litholytic agents. Briefly, cells were seeded in 96-well plates at a density of 1 × 104 cells/well in 100 μL complete medium (Wang F. et al., 2024). After 24 h of adhesion, litholytic agents were added at various concentrations and incubated for 48 h. Subsequently, 10 μL CCK-8 solution was added to each well, followed by additional incubation for 2 h at 37 °C. Absorbance was measured at 450 nm (reference wavelength: 650 nm) using a microplate reader (BioTek Synergy H1, Winooski, VT, United States), with cell viability calculated as: [OD (treatment)/OD (control)] × 100% (Cui et al., 2019).

### 2.6 Screening of targeted litholytic agents

Three categories of pharmaceutical agents were evaluated. All compounds were dissolved in deionized water at concentrations determined through preliminary testing. The solutions were then incubated with calcium oxalate stones under the experimental conditions described in the “*In Vitro* Stone Dissolution Assay.”

Crystal-targeting agents: citric acid monohydrate (≥99.5%; Solarbio C9861, China) at 1, 10, 100, 200, 300, 400, and 500 mg/mL; sodium polyphosphate (AR grade; Aladdin S165313, China) at 2.5, 5, 7.5, and 10 mg/mL; potassium permanganate (AR grade; Henan Huakai HKAR-6500, China) at 1:2,000 dilution (0.5 mg/mL) and 1:5,000 dilution (0.2 mg/mL); and magnesium citrate nonahydrate (AR grade; Aladdin M102711, China) at 1 and 2.5 mg/mL (saturated at 25 °C).

Matrix protein-targeting agents: trypsin (BR grade; Yuanye Bio S10032, China) at 0.05, 0.1, 1, and 10 mg/mL; and ammonium sulfate (99%; Macklin A801012, China) at saturated (639.2 mg/mL) and half-saturated (319.6 mg/mL) concentrations.

Microbiota-targeting agents: lysozyme (Ultra Pure grade; Solarbio L8120, China) at 0.0033, 0.0067, 1, 10, and 50 mg/mL; furazolidone (98%; Macklin F810004, China) at 1.25 and 2.5 mg/mL; levofloxacin (≥98%; Aladdin L157745, China) at 1, 2, and 4 mg/mL; cefixime (≥98%; Aladdin C343338, China) at 1 and 2 mg/mL; and piperacillin-tazobactam sodium (Hainan Tongyong Sanyang Pharmaceutical Co., Ltd., Haikou, China; National Drug Approval No. H19990188; Batch No. 240328-1) at 11.25, 22.5, 45, 67.5, and 90 mg/mL.

### 2.7 Design and optimization of multidimensional combinatorial formulations

A multidimensional combinatorial strategy was designed to explore synergistic litholytic effects, incorporating diverse drug classes and dynamic dose ratios. Primary litholytic agents (1-2 compounds) were selected based on preliminary drug screening results. The doses of adjunctive drugs were then dynamically adjusted to maintain the system pH within a strictly controlled range of 5.0–8.0. This pH regulation was achieved by titrating acidic or alkaline reagents within the formulation. Chemical equilibrium of the drug mixture was defined as the critical point where further addition of any drug component induced crystal precipitation or halted dissolution progress. The resulting solution at this equilibrium was designated as the “stock solution” and labeled with its specific formulation ID and pH value.

Initial combinatorial formulations, focused primarily on crystal dissolution, were evaluated using the *in vitro* Stone Dissolution Assay (refer to the section “*In Vitro* Stone Dissolution Assay” for details). Stone samples were incubated with the combinatorial solutions under controlled conditions (e.g., 37 °C water bath). Litholytic efficacy was quantified by measuring the percentage reduction in stone mass (dissolution rate) and qualitatively assessed by examining structural changes in the stones, including surface smoothness, integrity, and hardness. Formulations demonstrating superior dissolution efficacy (>80% mass reduction and favorable structural degradation) were advanced. Less effective formulations underwent iterative optimization, involving adjustments to drug dose ratios or the combination types themselves.

For the optimized crystal-dissolving formulations, agents effective against matrix proteins and microorganisms, identified in the “Screening of Effective Litholytic Agents” step, were incorporated. The modified formulations were then re-evaluated using the same *in vitro* stone dissolution protocol to assess their comprehensive litholytic effect.

The cytotoxicity of stock solutions with and without matrix-degrading and microbiota-targeting agents toward human urothelial cells (SV-HUC-1) was assessed. Each stock solution was serially diluted using a quartering method to achieve concentrations of 25%, 50%, 75%, and 100% (v/v). Cell viability after 48 h of exposure to these dilutions was measured using the CCK-8 assay. Formulations exhibiting >50% cell viability across the tested range were considered acceptable. Formulations showing significant cytotoxicity (viability <50% at higher concentrations) were further diluted to lower concentrations (10%, 20%, 30% v/v) and re-tested. The highest non-cytotoxic concentration (or concentration with minimal acceptable cytotoxicity) for each effective formulation was selected. The litholytic efficacy of these diluted solutions was then re-evaluated using the *in vitro* dissolution assay. Formulations exhibiting compromised dissolution efficacy upon dilution underwent further combinatorial adjustments and re-testing.

The final Multi-Target Compound Litholytic Solution (MTCLS) was selected based on the optimal balance between high dissolution efficacy (>50% mass reduction with significant structural degradation) and low cytotoxicity (cell viability comparable to or better than the control, e.g., citrate).

### 2.8 Comparison of stone dissolution efficacy: MTCLS vs. TCD

MTCLS was tested against trisodium citrate dihydrate (TCD, ACS grade, ≥99%, Aladdin S116314, China), the current gold standard, to compare its efficacy at dissolving CaOx (Robinson et al., 2020). All stones were initially weighed, and a selection within a narrow mass range (24.5–30.5 mg) was chosen for the study. The stones then underwent stratified randomization into groups of three so that each group contained one relatively large, one medium, and one small stone. This approach was adopted because a preliminary study showed that initial stone mass significantly affected the dissolution rate. Stones were placed in 5 mL of normal saline (NS),or MTCLS or TCD (200 mM) and incubated at 37 °C. For simplicity and to minimize confounding factors, a static rather than a flow model was used in this proof-of-concept study. Every 24 h, stones were carefully removed from solution, blotted with absorbent paper, and weighed; after measurement, samples were returned to their original containers and supplied with fresh medicinal solution. CT scans of the stones were performed before and after medication.

### 2.9 Micro-CT scanning and stone analysis

Micro computed tomography (micro-CT) scans were performed using a Viva CT80 scanner (SCANCO Medical AG, Switzerland) before and after dissolution experiments. The scanning parameters were set as follows: tube voltage 55 kVp, tube current 145 μA, and resolution 15.6 μm. Reconstruction, numerical analysis, and 3D rendering were conducted using the manufacturer’s dedicated SCANCO software suite. Identical parameters were employed for all scans.

Method for determining total stone volume and surface area:The total volume and surface area were calculated by performing three-dimensional analysis with a low binary threshold algorithm to separate the stone from air. Additionally, the CT values of the stone were determined using the five-point averaging method (all CT values were automatically computed by the SCANCO software). The maximum cross-sectional area of the stone was selected, and its longest diameter was divided into five equal segments for measurement, after which the mean CT value was calculated (Wei et al., 2019).

### 2.10 Animal experiment design

Thirty-six specific pathogen-free (SPF) Sprague-Dawley (SD) rats, each weighing between 230 and 270 g, were utilized in this study. Daily observations were conducted to monitor their general health status, focusing on mental alertness, food intake, urination patterns, and any abnormal reactions such as lethargy, hematuria, or vomiting throughout the treatment period.

Oral gavage groups (n = 6 per group): The negative control group received normal saline (NS, 0.9% NaCl). The Positive Control group was administered the TCD solution, serving as the reference standard. The Experimental Group received the Multi-Target Compound Litholytic Solution (MTCLS). Each group underwent once-daily oral gavage for seven consecutive days, with a dosage volume of 1.5 mL per 100 g of body weight.

Intravesical instillation groups (n = 6 per group): The negative control group received NS, while the positive control group was administered the TCD solution. The experimental group was given MTCLS. Rats were anesthetized using an intraperitoneal injection of pentobarbital sodium (40 mg/kg) dissolved in normal saline. A sterile, single-use epidural anesthesia catheter (Type F3-I, outer diameter 1.0 mm) was lubricated with sterile liquid paraffin and carefully inserted transurethrally into the bladder to a depth of approximately 2.5 cm. Gentle suprapubic pressure was applied to expel any residual urine. Subsequently, 0.5 mL of the assigned solution was instilled into the bladder through the catheter. The solution was retained for 1 h, during which the rats regained consciousness and spontaneously voided the instilled fluid. This procedure was conducted once daily for 7 consecutive days.

Sample Collection and Processing: 24 hours following the final administration, rats were deeply anesthetized with an intraperitoneal injection of sodium pentobarbital. Blood samples, approximately 4 mL from the gavage groups, were drawn from the abdominal aorta into Lithium heparin tubes. Within 30 min, the blood was centrifuged at 3,000 rpm for 15 min at 4 °C. The plasma obtained was aliquoted and stored at −80 °C for future analysis. Subsequently, the rats were euthanized through cervical dislocation while still under anesthesia. The stomachs from the gavage groups and bladders from the instillation groups were dissected and immediately fixed in a 4% paraformaldehyde (PFA) solution for at least 24 h before undergoing histological processing.

### 2.11 Liver and kidney function tests

Plasma alanine aminotransferase (ALT), aspartate aminotransferase (AST), blood urea nitrogen (BUN), and creatinine (Cr) were measured with commercially available assay kits (Jiancheng Bioengineering Institute, Nanjing, China) according to the manufacturers’ instructions to assess changes in hepatic and renal function.

### 2.12 H&E staining, mast cell, and immunohistochemistry detection

Paraffin sections were prepared from SD rat stomachs (n = 6) for hematoxylin and eosin (HE) staining, and from SD rat bladders (n = 6) for HE staining, mast cell staining, and immunohistochemistry. The specific procedures followed those reported previously (Shih et al., 2021; Zhao et al., 2025). The antibodies used in this study included rabbit polyclonal anti-Uroplakin III (1:200, WL05318, Wanleibio, China), rabbit polyclonal anti–E-Cadherin (1:200, WL00941, Wanleibio, China), rabbit polyclonal anti-TNFα (1:300, WL01896, Wanleibio, China), rabbit polyclonal anti-IL-1β (1:150, bs-25615R, Bioss, China), rabbit monoclonal anti-NLRP3 (1:100, ET160-93, Huaan, China), and horseradish peroxidase–conjugated goat anti-rabbit IgG (ready to use, HA1119, Huaan, China). HE and mast cell images were captured with a light microscope, and mast cells in the sections were counted under the same microscope. Immunohistochemical analysis was performed as previously described, and average optical density values were calculated with ImageJ software (Zhao et al., 2022; Yang et al., 2023).

### 2.13 Statistical analysis

Statistical analysis was conducted using GraphPad Prism 10.4.1 software. The Kolmogorov-Smirnov one-sample test evaluated the normality of the distribution of continuous variables prior to further comparisons. Normally distributed measurement data are presented as the mean ± standard deviation (M ± SD), and group comparisons were performed using *t* tests. Quantified data are expressed as frequency or percentage (%), with comparisons made using the chi-square test. Multigroup comparisons of means utilized either one-way ANOVA or the Kruskal–Wallis test, depending on the variance distribution, followed by *post hoc* comparisons using the Student-Newman-Keuls test. Bonferroni correction was applied for adjustment. A p-value of <0.05 was deemed statistically significant (ns: not significant, *P < 0.05, **P < 0.01, ***P < 0.001).

## 3 Results

### 3.1 Evaluation of targeted drug screening

#### 3.1.1 Evaluation of the litholytic efficacy of stone crystal dissolving drugs

**FIGURE 1:**
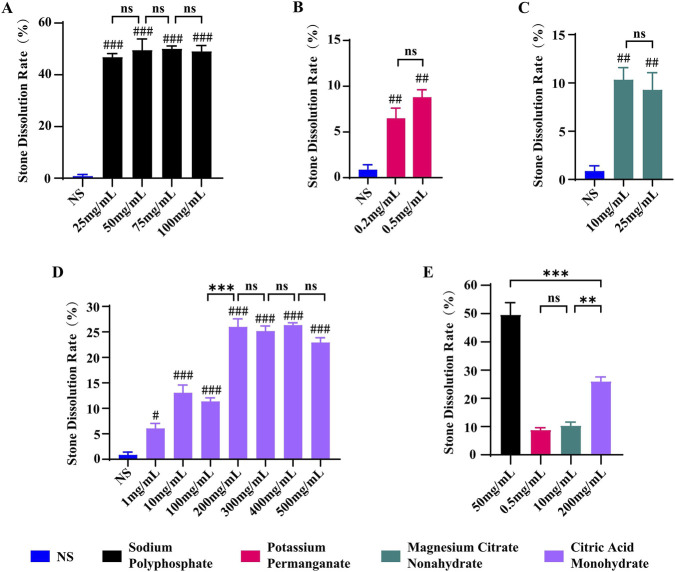
Evaluation of the dissolution effects of four targeted agents on calcium oxalate crystals. **(A)** Comparison of the dissolution effects of sodium polyphosphate at different concentrations; **(B)** Comparison of the dissolution effects of potassium permanganate at different concentrations; **(C)** Comparison of the dissolution effects of Magnesium Citrate Nonahydrate at different concentrations; **(D)** Comparison of the dissolution effects of citric acid monohydrate at different concentrations; **(E)** comparison of the dissolution effects of the four agents at their respective optimal concentrations. Data are presented as mean ± SD (*n* = 3). ^#^ *P* < 0.05, ^##^ *P* < 0.01, ^###^ *P* < 0.001 vs. NS group; ^ns^ *P >* 0.05, **P < 0.01, ***P < 0.001 between indicated groups.

We evaluated the *in vitro* dissolution of CaOx stone by four reagents—sodium polyphosphate, citric acid monohydrate, magnesium citrate nonahydrate, and potassium permanganate—across a range of concentrations. Compared to normal saline (NS), all tested reagents at the concentrations used resulted in significantly greater stone dissolution (p < 0.05). Sodium polyphosphate exhibited consistent dissolution efficacy across the concentration range tested, with no appreciable increase at higher concentrations (Figure 1A). In contrast, citric acid monohydrate showed a concentration-dependent increase in dissolution up to 200 mg/mL, beyond which further increases in concentration did not enhance the effect (Figure 1D). Magnesium citrate nonahydrate and potassium permanganate also demonstrated detectable dissolution activity (Figures 1B,C); however, their effects were substantially lower than those of sodium polyphosphate and citric acid monohydrate (Figure 1E). When compared directly, sodium polyphosphate produced the highest stone-dissolving effect among the four reagents, followed by citric acid monohydrate (Figure 1E).

#### 3.1.2 Evaluation of the litholytic efficacy of stone matrix protein degradation drugs

**FIGURE 2:**
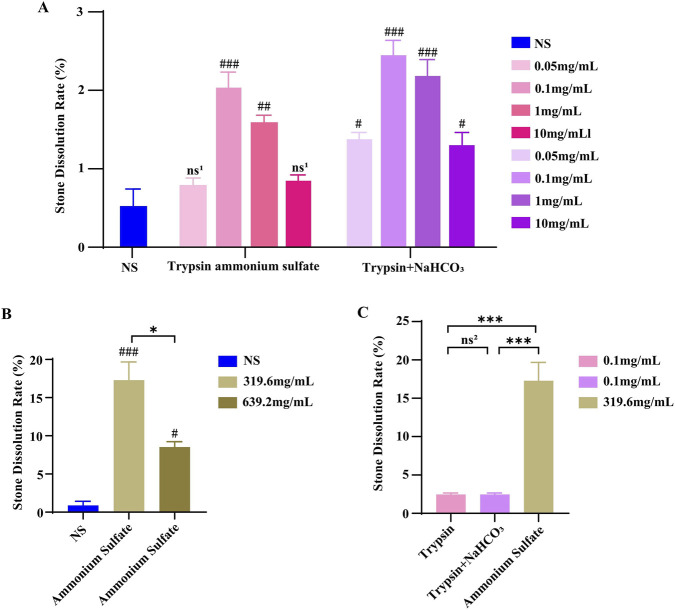
Evaluation of the dissolution effects of agents targeting matrix protein degradation. **(A)** Comparison of the dissolution effects of trypsin at different concentrations; **(B)** Comparison of the dissolution effects of ammonium sulfate at different concentrations; **(C)** Comparison of the dissolution effects of trypsin and ammonium sulfate at their respective optimal concentrations. In panels A and C, the concentration values in the legend indicate trypsin concentrations, and “+ NaHCO_3_” indicates the addition of sodium bicarbonate at the same concentration as trypsin. Data are presented as mean ± SD (*n* = 3). ^ns1^ *P >* 0.05, ^#^ *P* < 0.05, ^##^ *P* < 0.01, ^###^ *P* < 0.001 vs. NS group; ^ns2^ *P >* 0.05, *P < 0.05, ***P < 0.001 between indicated groups.

To investigate the degradation of matrix proteins in CaOx stones, we assessed the *in vitro* dissolution effects of trypsin and ammonium sulfate, at varying concentrations. Trypsin showed a concentration-dependent increase in dissolution up to 0.1 mg/mL, with no further increase or a decrease at higher concentrations (Figure 2A). At a fixed trypsin concentration, increasing the solution pH was associated with greater dissolution (Figure 2A). Ammonium sulfate also demonstrated a notable dissolving effect under the tested conditions (Figure 2B). When compared directly, ammonium sulfate resulted in greater dissolution than trypsin under the conditions evaluated (Figure 2C).

#### 3.1.3 Evaluation of the litholytic efficacy of microbial degradation drugs

**FIGURE 3:**
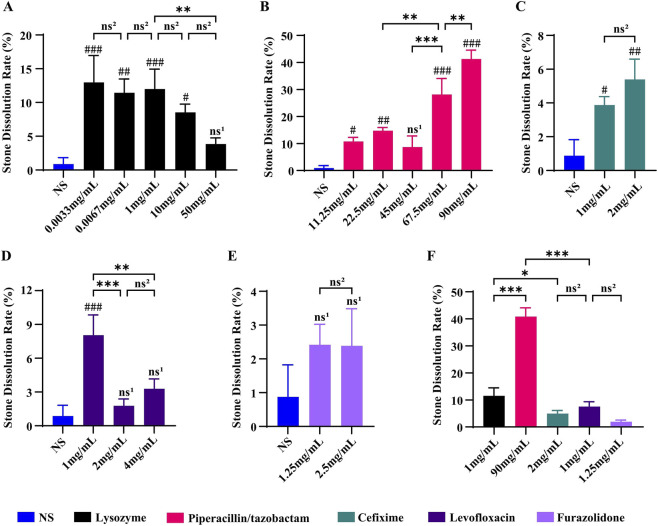
Evaluation of the dissolution effects of agents targeting microbial degradation. **(A)** Comparison of the dissolution effects of lysozyme at different concentrations; **(B)** Comparison of the dissolution effects of piperacillin/tazobactam at different concentrations; **(C)** Comparison of the dissolution effects of cefixime at different concentrations; **(D)** Comparison of the dissolution effects of levofloxacin at different concentrations; **(E)** Comparison of the dissolution effects of furazolidone at different concentrations; **(F)** Comparison of the dissolution effects of the five agents at their respective optimal concentrations. Data are presented as mean ± SD (*n* = 3). ^ns1^ *P >* 0.05, ^#^ *P* < 0.05, ^##^ *P* < 0.01, ^###^ *P* < 0.001 vs. NS group; ^ns2^ *P >* 0.05, *P < 0.05, **P < 0.01, ***P < 0.001 between indicated groups.

We assessed the *in vitro* dissolution effects of five antibacterial drugs, namely, lysozyme, cefixime, piperacillin/tazobactam, levofloxacin, and furazolidone, on calcium oxalate stones across a range of concentrations. Compared to NS, lysozyme, cefixime, piperacillin/tazobactam, and levofloxacin each resulted in certain dissolution effects, with statistically significant differences observed (Figures 3A–D, P < 0.05). In contrast, furazolidone did not show a statistically significant difference in dissolution effect compared to NS (Figure 3E, p > 0.05). At the concentrations tested, the dissolution effect of lysozyme was greater at lower concentrations than at higher concentrations (Figure 3A). In contrast, the dissolution effect of piperacillin/tazobactam increased with increasing concentration at the concentrations tested (Figure 3B). When compared directly under the conditions evaluated, piperacillin/tazobactam resulted in the greatest dissolution effect, followed by lysozyme (Figure 3F).

### 3.2 Design and optimization of multi-component formulation systems

Based on the targeted drug screening results, we initially prepared candidate solutions aimed at dissolving stone crystals. Citric acid monohydrate was used as the primary compound, with sodium polyphosphate optionally included, and the pH was adjusted using sodium carbonate, potassium carbonate, or a combination of both. These solutions were referred to as stock solutions, as outlined in Table 2. Each solution was then tested against calcium oxalate stones following the protocol detailed in “*in Vitro* Stone Dissolution Assay,” with normal saline serving as the negative control. Stone masses were kept between 10 and 20 mg. The stones were categorized by size and randomly assigned into 10 groups, corresponding to different drug combinations, labeled “A” through “I.” This categorization ensured that each group contained a relatively large, medium, and small stone, and that stone mass did not significantly differ among groups before dissolution.

**TABLE 2:** Candidate formulations for targeting calcium oxalate crystals.

Formulation ID	Citric acid monohydrate (g)	Sodium polyphosphate (g)	Sodium carbonate (g)	Potassium carbonate (g)	Deionized water (mL)	pH (stock solution)
A	6	—	—	5	30	6.09
B	6	—	5	—	30	6.65
C	6	—	2	3	30	6.30
D	6	3	—	4	30	5.34
E	3	6	—	2.5	30	5.82
F	6	3	4	—	30	5.45
G	3	6	2.5	—	30	5.66
H	6.5	3	3	3	30	6.86
I	3	6	1	2	30	6.78

**FIGURE 4:**
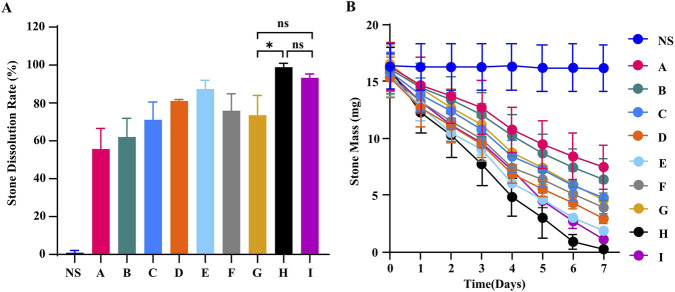
Evaluation of the stone dissolution effects of the combination formulations listed in Table 2. **(A)** Comparison of the 7-day stone dissolution rates among the candidate formulations. **(B)** Time course changes in residual stone mass during the 7-day treatment period. Data are presented as mean ± SD (*n* = 3). ^ns^ *P >* 0.05, *P < 0.05.

Figure 4A summarizes the 7-day stone dissolution effects of the solution combinations listed in Table 2. All tested combinations produced some degree of dissolution of calcium oxalate stones compared to the normal saline (NS). The time course of stone mass (Figure 4B) showed that combination “H” resulted in the least residual mass after day 3, whereas combination “A” resulted in the greatest residual mass. The NS control showed no significant change in stone mass over the 7-day period. Based on these observations, combination “H” was selected for further study. Four variant formulations were prepared by adding, respectively, ammonium sulfate, trypsin, piperacillin/tazobactam, and lysozyme to combination “H”; these were designated “H-1,” “H-2,” “H-3,” and “H-4” (Table 3). Dosages for each additive were set based on the results of the prior experiments (Figures 1–3).

**TABLE 3:** Candidate dual-target formulations against crystal and matrix or crystal and microorganism.

Formulation ID	Citric acid monohydrate (g)	Sodium polyphosphate (g)	Potassium carbonate (g)	Ammonium sulfate (g)	Trypsin (g)	Piperacillin-tazobactam sodium (g)	Lysozyme (g)	Deionized water (mL)	pH value (stock solution)
H-1	6.5	3	3	9.6	—	—	—	30	5.68
H-2	6.5	3	3	—	0.003	—	—	30	6.85
H-3	6.5	3	3	—	—	2.7	—	30	6.55
H-4	6.5	3	3	—	—	—	0.03	30	6.32

**FIGURE 5:**
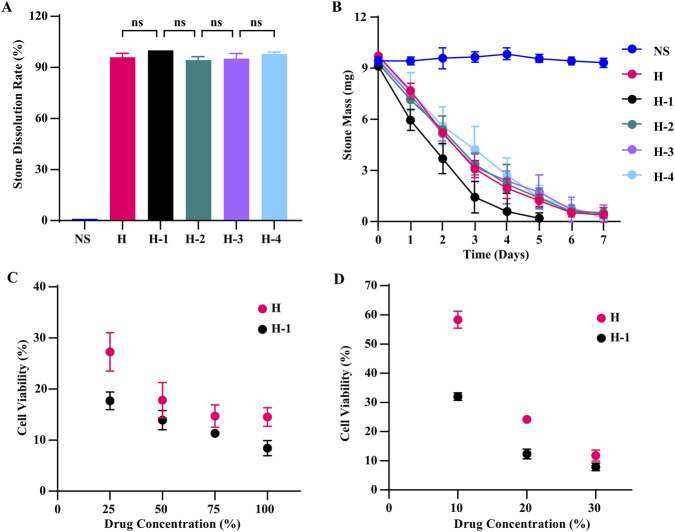
Comparison of dissolution efficacy of the combination formulations listed in Table 3 and safety assessment of selected formulations. **(A)** Comparison of the 7-day stone dissolution rates of the combination formulations. **(B)** Time course of residual stone mass during treatment with the combination formulations. **(C,D)** Comparison of the viability of human urothelial cells treated with different concentrations of formulations “H” and “H-1” (CCK-8 assay). Data are presented as mean ± SD (A,B: *n* = 3; C,D: *n* = 6). ^ns^ *P >* 0.05.

Combined drug solutions “H,” “H-1,” “H-2,” “H-3,” and “H-4” were incubated with calcium oxalate stones following the protocol described in the “*in Vitro* Stone Dissolution Assay.” Stone masses were maintained within a range of 9.0–9.8 mg, and all other conditions were unchanged. After 7 days of treatment, stones in all groups were nearly completely dissolved, with calculated dissolution rates approaching or reaching 100% (Figure 5A); no statistically significant differences were observed among the groups. With respect to the time course, drug “H-1” achieved complete dissolution by day 5 (Figure 5B), whereas the other agents required 7 days, suggesting a more rapid dissolution effect for “H-1” under the conditions tested.

Based on these findings, compound “H-1” was selected for further experiments. Both “H” and “H-1” were diluted using a 4-point method, with stock solution proportions of 25%, 50%, 75%, and 100%, and then subjected to the CCK-8 assay. The results (Figure 5C) showed that both drugs were associated with low cell survival rates in urothelial SV-HUC-1 cells across all concentrations tested, with 48-h survival rates below 30%. Under these conditions, “H-1” was associated with a lower survival rate than “H.” Accordingly, the drug concentrations were adjusted to 10%, 20%, and 30%, and the CCK-8 assay was repeated. The results (Figure 5D) indicated that at equivalent concentrations, “H-1” continued to be associated with lower cell survival rates compared to “H.”

Based on the results of the two CCK-8 experiments, and given the low cell survival rates associated with ammonium sulfate in SV-HUC-1 cells, the “H-1” protocol was not pursued further. Because “H” was associated with relatively higher cell survival rates at the 10% concentration, subsequent experiments were conducted using this concentration. Previous experiments showed no statistically significant differences in the dissolution effect of lysozyme on stones within the concentration range of 0.0033–1.0 mg/mL (Figure 3A). In addition, to avoid potential interference between lysozyme and trypsin, a concentration of 0.1 mg/mL was selected for further experiments. To ensure adequate dissolution of all components during preparation, solutions were prepared directly at the 10% diluted concentration of “H” (Table 4).

**TABLE 4:** Proposed multi-target formulations for comprehensive stone dissolution.

Formulation ID	Citric acid monohydrate (g)	Sodium polyphosphate (g)	Sodium carbonate (g)	Potassium carbonate (g)	Trypsin (g)	Piperacillin-tazobactam sodium (g)	Lysozyme (g)	Deionized water (mL)	pH value
H-I	2.17	1	1	1	—	—	—	100	7.02
H-II	2.17	1	1	1	0.01	9	—	100	7.35
H-III	2.17	1	1	1	0.01	—	0.01	100	7.56
H-IV	2.17	1	1	1	0.01	9	0.01	100	7.50

**FIGURE 6:**
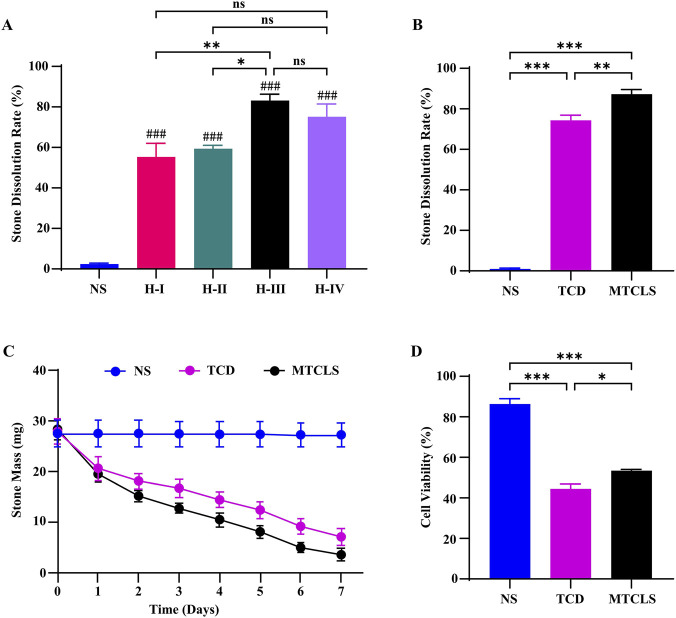
Evaluation of dissolution efficacy and cytotoxicity of MTCLS. **(A)** Comparison of the 7-day stone dissolution rates of the candidate multi-target formulations listed in Table 4. **(B)** Comparison of dissolution rates between MTCLS and the current gold standard, trisodium citrate dihydrate (TCD). **(C)** Time course of residual stone mass during treatment with MTCLS and TCD. **(D)** Comparison of the effects of MTCLS and TCD on the viability of human urothelial cells (CCK-8 assay). Data are presented as mean ± SD (A–C: *n* = 3; D: *n* = 6). ^###^ *P* < 0.001 vs. NS group; ^ns^ *P >* 0.05, *P < 0.05, **P < 0.01, ***P < 0.001 between indicated groups.

*In vitro* experiments to assess the stone dissolution of the “H-I to H-IV” drugs, as listed in Table 4, were conducted under previously described conditions. The stone mass was maintained within a range of 24.5–30.5 mg. The dissolution rates observed 7 days post-medication are depicted in Figure 6A. While no statistical difference was found between “H-III” and “H-IV,” a significant difference was noted between “H-III” and both “H-I” and “H-II.” However, “H-IV” did not show a statistically significant difference when compared to “H-I” and “H-II.” Based on these results, “H-III” was selected as the Multi-Target Compound Litholytic Solution (MTCLS).

We compared MTCLS with the current gold standard, Citrate (Trisodium Citrate Dihydrate, TCD), to evaluate *in vitro* stone dissolution effects (for details, “Comparison of Stone Dissolution Efficacy: MTCLS vs. TCD”). The results of the 7-day stone dissolution rates are presented in Figure 6B, demonstrating that MTCLS is statistically more effective than TCD. Figure 6C illustrates the change in stone mass over time.

We subsequently performed a safety comparison between MTCLS and TCD using the CCK-8 experiment (for detailed methodology, the “CCK-8 assay” section). The results, presented in Figure 6D, indicate that while the cell survival rate with MTCLS was lower than with normal saline, it was higher than with MTCLS vs. TCD. This difference was statistically significant, suggesting that MTCLS has a relatively high safety profile.

### 3.3 *In vivo* safety assessment of MTCLS in animal models

Building on prior results, we conducted animal experiments to further verify the biological safety of MTCLS. The drug was administered via gavage and bladder perfusion, using NS as the negative control and TCD solution as the positive control.

**FIGURE 7:**
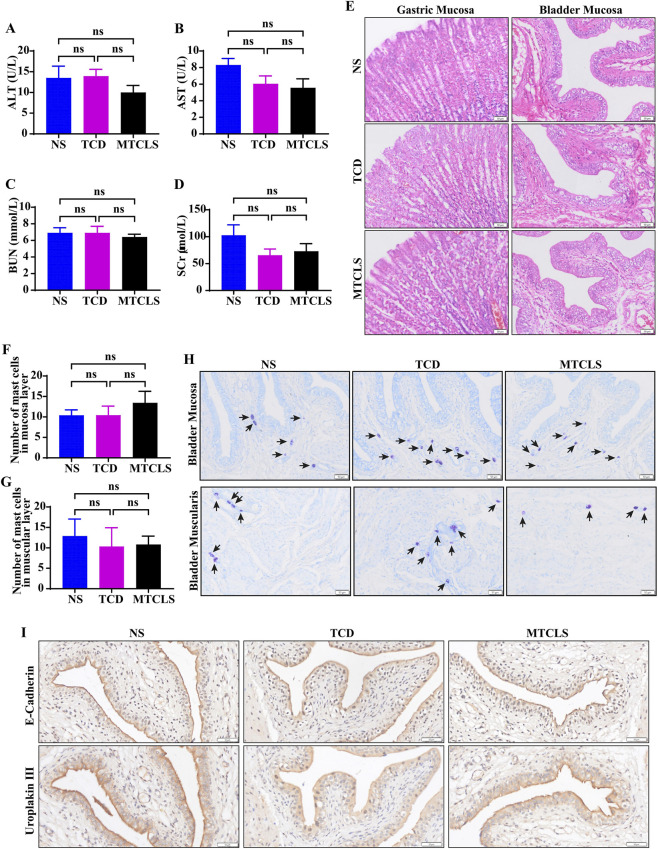
Systemic and local safety evaluation of MTCLS in a rat model. **(A–D)** Serum biomarkers of liver and kidney function: **(A)** alanine aminotransferase (ALT); **(B)** aspartate aminotransferase (AST); **(C)** blood urea nitrogen (BUN); **(D)** serum creatinine (SCr). **(E)** Representative H&E-stained sections of gastric and bladder tissues (100x). **(F–H)** Mast cell infiltration assessment in bladder tissue: **(H)** representative toluidine blue staining (100x)**; (F)** quantitative analysis in the mucosa; **(G)** quantitative analysis in the muscular layer. **(I)** Evaluation of epithelial integrity markers: representative images of E-Cadherin and Uroplakin III expression (200x). Scale bars = 50 µm (applies to all panels). Data are presented as mean ± SD (*n* = 6). ^ns^ *P >* 0.05.

Safety assessment of the gavage group: Seven days post-gavage, blood was collected from the inferior vena cava for hepatic/renal function analysis. Concurrently, gastric tissues were processed for H&E staining to evaluate the impact of MTCLS on gastric tissues. Key findings: Biochemical indices showed no significant differences in ALT, AST, BUN, or SCr among groups (p > 0.05, Figures 7A–D). Histopathological analysis revealed preserved gastric architecture across all groups, with no evidence of inflammation, edema, or epithelial damage (Figure 7E).

**FIGURE 8:**
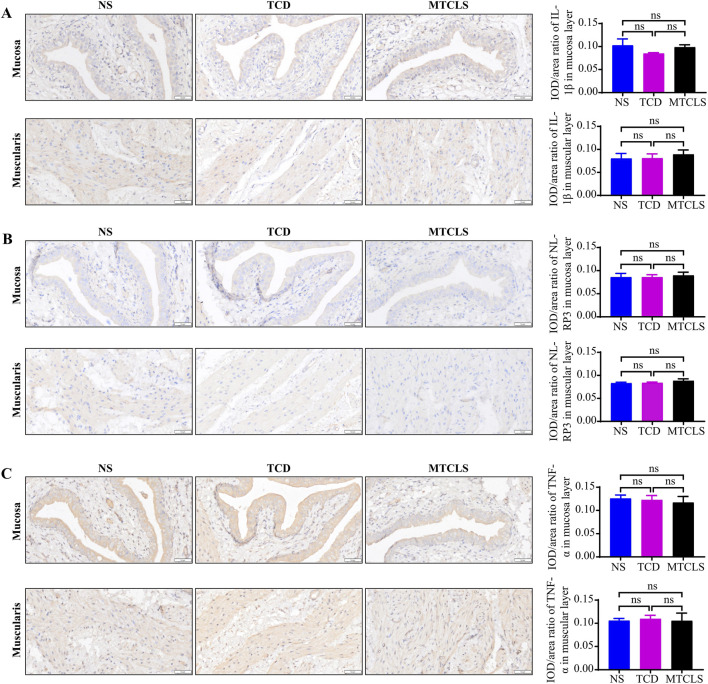
Immunohistochemical analysis of inflammatory cytokines in the bladder of SD rats. **(A)** Representative images of IL-1β expression and quantitative analysis in the mucosa and muscular layer. **(B)** Representative images of NLRP3 expression and quantitative analysis in the mucosa and muscular layer. **(C)** Representative images of TNF-α expression and quantitative analysis in the mucosa and muscular layer. Images were acquired at ×200 magnification. Scale bars = 50 µm (applies to all panels). Data are presented as mean ± SD (*n* = 6). ^ns^ *P >* 0.05.

Safety assessment of the bladder perfusion group: Seven days post-perfusion, bladder tissues were collected for H&E staining, mast cell staining, and immunohistochemical staining to evaluate the drug’s impact on the tissues. HE staining results indicated that, compared to the NS and TCD groups, the bladder morphology in the MTCLS group remained largely intact, with no significant epithelial edema or injury observed (Figure 7E). Mast cell staining revealed minimal infiltration across all groups (Figure 7H), with mast cells primarily located around blood vessels and scarcely present in non-vascular areas. Statistical analysis showed no significant differences among the groups (Figures 7F,G), thus excluding the possibility of pathological mast cell infiltration. To assess the integrity of the bladder urothelium, we performed immunohistochemical staining on paraffin sections using E-Cadherin and Uroplakin III. The results demonstrated that the urothelial continuity in the NS group, TCD group, and MTCLS group remained intact, with no apparent defects observed (Figure 7I). To gain deeper insights into the inflammatory state of bladder tissue, we conducted immunohistochemical staining for IL-1β, NLRP3, and TNF-α. The findings indicated no significant differences in the expression levels of IL-1β, NLRP3, and TNF-α between the MTCLS group and the control groups (NS group and TCD group) (Figures 8A–C). In conclusion, these findings suggest that the MTCLS does not exhibit any significant toxic or side effects on liver and kidney functions, gastric tissues, or bladder tissues.

### 3.4 Evaluation of MTCLS litholytic efficacy by micro-CT scanning

**FIGURE 9:**
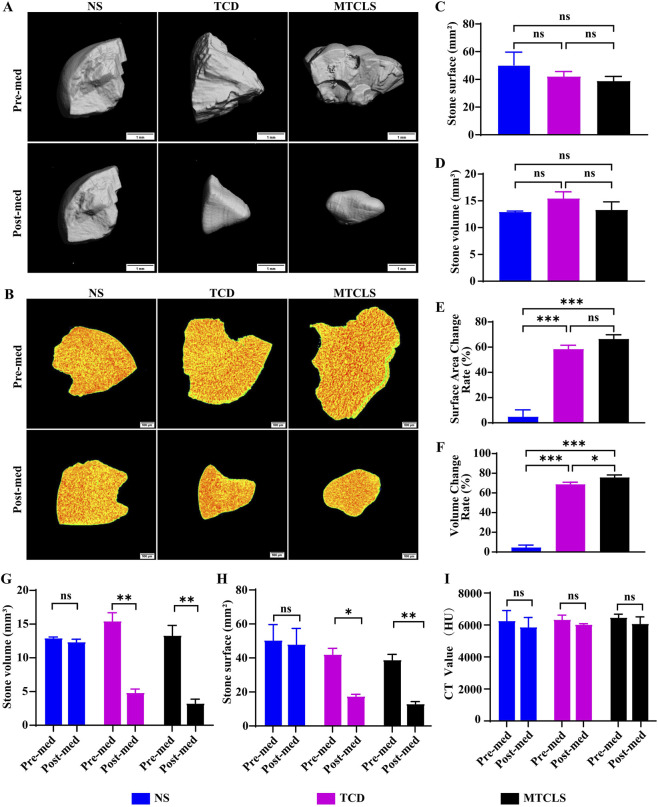
Micro-CT scanning and analysis of stones before and after MTCLS treatment. **(A)** Three-dimensional surface reconstruction of stones pre-medication (pre-med) and post-medication (post-med). Scale bar = 1 mm. **(B)** Cross-sectional views showing internal density distribution. Color gradient: red, high density; yellow, medium density; green, low density. Scale bar = 500 μm. **(C)** Comparison of surface area among groups pre-med. **(D)** Comparison of volume among groups pre-med. **(E)** Comparison of the rate of change in surface area among groups. **(F)** Comparison of the rate of change in volume among groups. **(G)** Comparison of volume pre-med and post-med within each group. **(H)** Comparison of surface area pre-med and post-med within each group. **(I)** Comparison of CT value pre-med and post-med within each group. Data are presented as mean ± SD (*n* = 3). ^ns^ *P >* 0.05, *P < 0.05, **P < 0.01, ***P < 0.001.

To systematically evaluate the effect of the MTCLS, we performed micro-CT scans on the stones both before and after treatment. Three-dimensional reconstructions of these scans (Figure 9A) revealed that the surface structure of stones in the normal saline (NS) group remained largely unchanged. In contrast, stones in the MTCLS and TCD groups showed significant surface passivation and rounding post-treatment, with a notable decrease in volume (Figure 9A). Calculations of the volume and surface area for each group indicated no statistically significant differences before treatment, confirming comparability (Figures 9C,D). Post-treatment, the NS group showed no significant changes in surface area or volume, whereas both the MTCLS and TCD groups experienced significant reductions in these metrics (Figures 9G,H). We assessed the change rates in surface area and volume of the stones before and after medication. The surface area change rate was calculated as [(surface area before medication − surface area after medication)/surface area before medication × 100%], and the volume change rate was calculated similarly [(volume before medication − volume after medication)/volume before medication × 100%]. Compared with the NS group, both the TCD and MTCLS groups showed statistically significant differences in surface area and volume change rates (Figures 9E,F). Compared with the TCD group, the MTCLS group did not differ significantly in surface area change rate (Figure 9E) but did show a statistically significant difference in volume change rate (Figure 9F). To better understand the internal structural changes in the calculi, we calculated the CT value of their maximum section. The findings revealed no significant alterations in the internal structure of the calculi before and after medication (Figures 9B,I). These results suggest that MTCLS primarily affects the stone’s surface, gradually dissolving it from the outside inward, without directly acting on the stone’s interior.

## 4 Discussion

In this study, we proposed a Multi-Target Compound Litholytic Solution (MTCLS) as a potential alternative to citrate and evaluated its *in vitro* dissolution effects on calcium oxalate (CaOx) stones, as well as its preliminary biosafety *in vitro* and *in vivo*. The main findings are as follows: sodium polyphosphate, citric acid monohydrate, trypsin, ammonium sulfate, lysozyme, and piperacillin/tazobactam each showed varying degrees of dissolution effects on CaOx stones *in vitro*. Following continuous compatibility optimization, the MTCLS formulation (containing sodium polyphosphate, citric acid monohydrate, trypsin, lysozyme, sodium carbonate, and potassium carbonate) was obtained. Compared with citrate (TCD), MTCLS exhibited greater dissolution efficacy *in vitro* and demonstrated favorable biosafety in a 7-day animal study.

Targeted drug screening results reveal that both citric acid monohydrate and sodium polyphosphate effectively dissolve calcium oxalate stones, with sodium polyphosphate demonstrating significantly higher efficacy (Figure 1). This finding aligns with the theoretical chelation mechanisms of these substances, though their efficacies differ. Citric acid monohydrate, a small organic acid, dissolves CaOx stones by forming soluble complexes with calcium ions (Ca^2+^) through its carboxyl groups (Shanthil et al., 2020). In contrast, sodium polyphosphate’s superior performance is due to its robust, multi-level synergistic mechanism. As a long-chain polymer, it results in a stronger affinity for Ca^2+^ and faster chelation kinetics (Deshwal et al., 2024; Kim et al., 2025), which more effectively disintegrates the crystal structure. Furthermore, the stability of calcium oxalate stones relies on an organic matrix formed by protein cross-linking through “calcium bridges” (Wesson, 2025). Sodium polyphosphate not only efficiently chelates Ca^2+^ within the crystals (Kim et al., 2025) but also disrupts these calcium bridges (Deshwal et al., 2024), leading to the complete loosening of the stone’s organic framework and the disintegration of its physical structure (Deshwal et al., 2024; Wesson, 2025).

Our further experiments demonstrated that the combined use of citric acid monohydrate and sodium polyphosphate, along with appropriate amounts of sodium carbonate and potassium carbonate, produces a superior stone dissolution effect compared to any single agent (Figure 4). This suggests that combination therapy can achieve synergistic enhancement through multiple mechanisms. Additionally, sodium carbonate and potassium carbonate primarily adjust the environmental pH and supplement potassium ions. This environment not only significantly enhances the efficiency of the chelation reaction but may also directly micro-etch the surface of calcium oxalate crystals, creating a chemical environment unfavorable for stone stability.

The targeted drug screening results indicated that ammonium sulfate at half-saturation concentration dissolved matrix proteins in calcium oxalate stones approximately twice as effectively as at saturation concentration and significantly better than trypsin (Figure 2). Although the exact mechanism by which ammonium sulfate dissolves stones is unclear, it is known that it cannot directly react with calcium oxalate crystals according to chemical principles and equilibrium theory. We hypothesize that its primary mechanism involves promoting the disintegration of stone crystals by degrading matrix proteins. Previous studies have shown that the S100 protein family dissolves well in fully saturated ammonium sulfate under neutral conditions (Messana et al., 2023). However, our study found that a half-saturated ammonium sulfate solution was more effective at dissolving stones than a fully saturated solution. We speculate that this half-saturated solution may target other stone matrices in addition to the S100 protein family, enhancing stone dissolution. Based on this finding, we incorporated ammonium sulfate into a multi-target stone-dissolving solution to more effectively dissolve calcium oxalate stones (Table 3). This solution, which includes ammonium sulfate, demonstrated a significantly better dissolution effect on CaOx stones compared to solutions containing trypsin (Figure 5B). Unfortunately, in subsequent cell safety detection experiments, the solution containing ammonium sulfate exhibited high toxicity to human urothelial cells (Figures 5C,D). Consequently, we discontinued further research on ammonium sulfate. For matrix protein degradation drugs, we ultimately selected trypsin. This alkaline protease demonstrates optimal enzymatic activity in a pH range from neutral to alkaline (Biškauskaitė et al., 2024), aligning with our findings that the dissolution effect of calculi was more pronounced after adding sodium bicarbonate (Figure 2A). Trypsin is known to specifically cleave the carboxyl terminals of arginine and lysine in protein polypeptide chains (Liu et al., 2020; Wang et al., 2020; Neukam et al., 2024). Proteins such as S100A8, S100A9 (Stephan and Nolan, 2016), osteopontin (Christensen et al., 2023), haptoglobin (Kozlik et al., 2022), and histones (Siddiqui et al., 2021) in the stone matrix are rich in arginine and/or lysine, and may therefore be susceptible to degradation by trypsin.

The results from targeted drug screening reveal that lysozyme and piperacillin-tazobactam sodium both facilitate the dissolution of calcium oxalate stones by targeting microorganisms within the stones. However, they exhibit distinctly different dose-response relationships (Figures 3A,B), with piperacillin-tazobactam sodium demonstrating a more pronounced overall effect (Figure 3F). This finding highlights the varying effectiveness and limitations of antibacterial strategies in the complex microenvironment of stones. Notably, lysozyme’s effectiveness decreases as its concentration increases (Figure 3A). Research indicates that lysozyme targets the peptidoglycan in the cell walls of both Gram-positive and Gram-negative bacteria, primarily affecting Gram-positive bacteria (Ferraboschi et al., 2021). We speculate that at high concentrations, lysozyme rapidly lyses Gram-positive bacteria on the stone surface, releasing internal contents like DNA and proteins. These substances may bind to the stone matrix components, forming a viscous “biological debris layer” that hinders further drug action, creating a self-inhibiting “barrier effect.” Conversely, piperacillin-tazobactam sodium’s effectiveness increases with concentration (Figure 3B), aligning with classic antibiotic pharmacology. As a broad-spectrum β-lactam compound, it targets both Gram-positive bacteria (e.g., enterococci and staphylococci) and Gram-negative bacteria (e.g., *Escherichia* in the phylum Proteobacteria) (Sartelli et al., 2014; Arain et al., 2023), thereby more thoroughly disrupting the biofilm structure supported by diverse microorganisms. As its concentration rises, its bactericidal effect enhances, avoiding the physical barrier issue seen with lysozyme, thus exhibiting a positive dose-response relationship.

To identify the optimal microbial degradation drugs, we developed several compatibility schemes (Table 4) and systematically evaluated them, including H-I to H-IV, through *in vitro* stone dissolution experiments. Results indicated that stone dissolution efficacy improved progressively from the basic chemical chelating agent combination (H-I) to the composite formula with trypsin and lysozyme (H-III) (Figure 6A). The difference between H-III and H-I was statistically significant (*P* < 0.05), suggesting that the combination of trypsin and lysozyme enhances chemical dissolution to some extent. Under alkaline conditions, the β-lactam ring of piperacillin is hydrolyzed and destroyed (Li et al., 2023), compromising its antibacterial activity. Consequently, in formula H-II (a weakly alkaline environment with pH 7.45), the antibacterial effect of piperacillin tazobactam sodium was not fully realized, resulting in lower stone dissolution efficacy compared to H-III (Figure 6A). This suggests that lysozyme plays a more crucial role in the mixed solution than piperacillin tazobactam sodium. Although H-IV (containing both antibiotics and lysozyme) showed numerically better results than H-II, it was less effective than H-III, with no statistically significant difference. Based on these findings, we preliminarily selected H-III as the target stone dissolution solution for several reasons: First, adhering to the optimization principle of “simplest and effective,” H-III achieves greater efficacy without high-dose antibiotics, potentially avoiding potential instability and cost increases from redundant components. Second, the chemical environment of H-III (pH 7.06) is milder, which may offer better compatibility and stability than H-IV (pH 7.50). Finally, from a clinical translation perspective, H-III may offer higher safety and economic benefits while maintaining potency. Thus, the selection of H-III was based on statistically significant differences in dissolution efficacy and our consideration of component compatibility, whereas its stability and cost-effectiveness are considered anticipated benefits requiring further validation.

This study conducted a comparative analysis between H-III and the current gold standard, citrate. The results indicated that H-III had significantly greater stone dissolution effect compared to TCD (Figures 6B,C), and it exhibited slightly lower toxicity to urothelial cells (Figure 6D). Consequently, we identified H-III as the Multi-Target Compound Litholytic Solution (MTCLS). In the 7-day animal study, MTCLS was not associated with significant changes in liver and kidney function, nor in gastric and bladder tissues (Figures 7, 8), suggesting acceptable short-term biosafety under the conditions tested.

The components of MTCLS include trypsin, lysozyme, sodium carbonate, potassium carbonate, sodium polyphosphate, and citric acid monohydrate. When administered orally, the effective concentration of trypsin and lysozyme reaching the urinary system is significantly reduced due to gastrointestinal metabolism. Trypsin readily denatures and loses activity in gastric juice, with the rate of denaturation increasing as pH decreases (Mahmoud El-Sayed Ali and Pearson, 2017). Lysozyme exhibits optimal activity at a pH range of approximately 5.3–6.4 (Shi et al., 2022), and it denatures and loses activity at pH levels below 2 (Heng et al., 2006) or above 7 (Guo et al., 2018). Consequently, the low pH of gastric acid may, to some extent, lead to loss of lysozyme activity, and pepsin may also affect its activity. Even with enteric coating to protect the enzymes until they reach the intestine, trypsin has almost no ability to cross the intact mucosal barrier and is barely absorbed under normal physiological conditions (Schmid-Schönbein, 2016). Orally administered lysozyme also shows poor stability and low bioavailability in the gastrointestinal tract (Khongkow et al., 2023). Therefore, to achieve therapeutic effects, trypsin and lysozyme would require specialized formulations or non-oral routes of administration.

Based on our experimental results, we hypothesize that the six components in MTCLS exert a superior dissolution effect on calcium oxalate stones when present together. In this study, micro-CT results showed that after MTCLS treatment, stone volume and surface area were significantly reduced, while CT values remained largely unchanged, suggesting that the dissolution effect of MTCLS on calcium oxalate stones may primarily occur from the outside inward. The proposed mechanism is as follows: citric acid monohydrate and sodium polyphosphate synergistically chelate calcium ions, efficiently disrupting the crystal structure of the outer layer of calcium oxalate stones and thereby exposing the embedded matrix proteins and microorganisms. Subsequently, trypsin degrades the matrix proteins, while lysozyme targets microorganisms, weakening the crystal connections. This process allows citric acid monohydrate and sodium polyphosphate to further chelate calcium ions, accelerating the dissolution of stone crystals. Through this cyclical mechanism, complete stone dissolution may be achieved. Considering this proposed mechanism and the pharmacokinetic challenges associated with trypsin and lysozyme, local administration of MTCLS may be a more suitable approach. For example, for recurrent calcium oxalate stones or residual fragments after ureteroscopic lithotripsy, retrograde ureteral catheterization can be used to achieve local perfusion for stone dissolution. For residual stones after percutaneous nephrolithotomy, local perfusion through the nephrostomy tract may be employed. Compared with oral administration, this localized approach may enhance local drug concentration while reducing systemic side effects associated with drug metabolism.

Although the MTCLS developed in this study showed certain dissolution potential *in vitro* and demonstrated some statistically significant advantages over citrate, with favorable short-term biosafety, certain limitations should be acknowledged. Firstly, due to technical constraints, this study only assessed the *in vitro* dissolution effect of MTCLS on calcium oxalate stones, and a suitable *in vivo* animal model for stone dissolution was not established, making it impossible to fully replicate the complex dynamics of the urinary system *in vivo*, such as continuous urine flow and tissue-stone interactions. In addition, other potential effects (such as inhibition of crystal growth or anti-inflammatory activity) were not evaluated. Therefore, the actual efficacy of this formulation under physiological conditions, as well as any broader therapeutic effects, remain to be further investigated. Secondly, the *in vivo* safety assessment was limited to a 7-day observation period; therefore, the long-term safety of MTCLS requires further investigation. Thirdly, the synergistic effects among the multiple components in MTCLS represent a hypothesized mechanism, and the exact mechanism of action still needs to be clarified in future studies. In summary, MTCLS cannot yet be considered an ideal therapy for calcium oxalate stones. Future research will focus on validating its *in vivo* dissolution efficacy and long-term safety, as well as further elucidating its mechanism of action on this basis to support potential clinical translation.

## 5 Conclusion

In this study, we developed a novel Multi-Target Compound Litholytic Solution (MTCLS). *In vitro* experiments demonstrated that MTCLS exhibited greater dissolution efficacy for calcium oxalate stones compared to conventional citrate therapy. In a 7-day animal study, MTCLS showed acceptable short-term (7-day) biosafety. Based on its component functions, we hypothesize that the dissolution effect of MTCLS may involve a combination of chemical chelation, enzymatic digestion, and antibacterial activity, though direct evidence for these mechanisms requires further investigation. The results of this study suggest that MTCLS is a promising multi-target candidate and a potential alternative to citrate for further investigation, offering a new perspective for the development of litholytic agents for calcium oxalate stones.

## Data Availability

The raw data supporting the conclusions of this article will be made available by the authors, without undue reservation.

## References

[B1] Al-Bayati M. Samarasinghe S. (2022). Biofilm and gene expression characteristics of the carbapenem-resistant Enterobacterales, *Escherichia coli* IMP, and *Klebsiella pneumoniae* NDM-1 associated with common bacterial infections. Int. J. Environ. Res. Pub. Health 19, 4788. 10.3390/ijerph19084788 35457654 PMC9024719

[B2] Alshareef A. Ahmed O. A. S. Abudrea M. B. Elkhadar A. A. M. Lieske J. C. (2025). Risk of CKD and ESRD in patients with kidney stones: a systematic review and meta-analysis with 7,045,880 participants. J. Am. Soc. Nephrol. 36 (10S). 10.1681/ASN.2025m3899a3v

[B3] Arain S. Khalawi F. Parakkal S. A. AlHamad H. S. Thorakkattil S. A. Alghashmari F. (2023). Drug utilization evaluation and impact of pharmacist interventions on optimization of piperacillin/tazobactam use: a retrospective analysis and prospective audit. Antibiot. (Basel, Switzerland) 12, 1192. 10.3390/antibiotics12071192 37508288 PMC10376400

[B4] Bargagli M. Scoglio M. Howles S. A. Fuster D. G. (2025). Kidney stone disease: risk factors, pathophysiology and management. Nat. Rev. Nephrol. 21, 794–808. 10.1038/s41581-025-00990-x 40790363

[B5] Biškauskaitė R. Lee W. C. Valeika V. (2024). Crude proteolytic enzyme from Bacillus halodurans BCRC 910501 and its application in leather processing. Heliyon 10, e35842. 10.1016/j.heliyon.2024.e35842 39229517 PMC11369431

[B6] Cerón J. J. Ortín-Bustillo A. López-Martínez M. J. Martínez-Subiela S. Eckersall P. D. Tecles F. (2023). S-100 proteins: basics and applications as biomarkers in animals with special focus on calgranulins (S100A8, A9, and A12). Biol. (Basel) 12, 881. 10.3390/biology12060881 37372165 PMC10295460

[B7] Chen G. Ren H. (2022). The development and application of a triage system for urolithiasis during COVID-19. World J. Urol. 40, 577–583. 10.1007/s00345-021-03871-7 34762172 PMC8581286

[B8] Chen X. W. Zheng Y. Y. Ouyang J. M. (2023). Sulfated Undaria pinnatifida polysaccharide promotes endocytosis of nano-calcium oxalate dihydrate by repairing subcellular organelles in HK-2 cells. Antioxidants (Basel, Switzerland) 12, 1015. 10.3390/antiox12051015 37237881 PMC10215875

[B9] Christensen B. Nielsen N. R. Sørensen M. R. Jacobsen L. N. Ostenfeld M. S. Sørensen E. S. (2023). Naturally occurring N-Terminal fragments of bovine milk osteopontin are transported across models of the intestinal barrier. Biomedicines 11, 893. 10.3390/biomedicines11030893 36979872 PMC10045268

[B10] Crivelli J. J. Oerline M. K. Maalouf N. M. Hsi R. S. Krampe N. A. Smith K. B. (2026). Adherence to potassium citrate, changes in 24-hour stone risk parameters, and recurrent kidney stone events. Kidney360. 10.34067/KID.0000001157 41774501 PMC13450965

[B11] Cui L. Jiang X. Zhang C. Li D. Yu S. Wan F. (2019). Ketamine induces endoplasmic reticulum stress in rats and SV-HUC-1 human uroepithelial cells by activating NLRP3/TXNIP aix. Biosci. Rep. 39, BSR20190595. 10.1042/BSR20190595 31652453 PMC6811748

[B12] Dai J. C. Maalouf N. M. Hill K. Antonelli J. A. Pearle M. S. Johnson B. A. (2023). Alkali citrate content of common over-the-counter and medical food supplements. J. Endourol. 37, 112–118. 10.1089/end.2022.0274 35972746

[B13] Dawson C. H. Tomson C. R. (2012). Kidney stone disease: pathophysiology, investigation and medical treatment. Clin. Med. (London, England) 12, 467–471. 10.7861/clinmedicine.12-5-467 23101150 PMC4953772

[B14] Deshwal G. Fenelon M. Gómez-Mascaraque L. G. Huppertz T. (2024). Influence of citrate- and phosphate-based calcium sequestering salts on the disruption of casein micelles. Food Hydrocollids 153, 109970. 10.1016/j.foodhyd.2024.109970

[B15] Fan Q. X. Gong S. Q. Hong X. Z. Feng X. M. Zhang F. J. (2020). Clinical-grade Garcinia cambogia extract dissolves calcium oxalate crystals in drosophila kidney stone models. Eur. Rev. Med. Pharmacol. Sci. 24, 6434–6445. 10.26355/eurrev_202006_21542 32572941

[B16] Ferraboschi P. Ciceri S. Grisenti P. (2021). Applications of lysozyme, an innate immune defense factor, as an alternative antibiotic. Antibiot. (Basel, Switzerland) 10, 1534. 10.3390/antibiotics10121534 34943746 PMC8698798

[B17] Ferraro P. M. Taylor E. N. Curhan G. C. (2024). 24-Hour urinary chemistries and kidney stone risk. Am. J. Kidney Dis. 84, 164–169. 10.1053/j.ajkd.2024.02.010 38583757 PMC13170619

[B18] Guo W. Zhou S. Yu Y. Wu J. Chang C. (2018). Antimicrobial stability of lysozyme under different pH,temperature and metal ions. Sci. Technol. Food Industry 39, 40–44.

[B19] Han X. Zhang Z. Yao P. Yang X. (2025). Infrared spectroscopic analysis of urinary stone composition. Actas Urol. Espanolas 49, 501810. 10.1016/j.acuroe.2025.501810 40633662

[B20] Heng H. Ke W. Ji K. (2006). The raman spectral analysis of lysozyme solution treated with acid at various pH. J. Nanjing Normal Univ. (Natural Science) 29, 48–51.

[B21] Hershenhouse J. S. Hom B. M. Bajakian T. H. Nguyen M. M. (2025). Palatability preferences of non-capsular potassium citrate alternatives in healthy non-stone-forming adults. J. Endourol. 39, 1262–1268. 10.1177/08927790251387360 41169015

[B22] Khongkow M. Rimsueb N. Jantimaporn A. Janyaphisan T. Woraprayote W. Visessanguan W. (2023). Cationic liposome of hen egg white lysozyme for enhanced its stability,activity and accessibility in gastro-intestinal tract. Food Biosci. 53, 102470. 10.1016/j.fbio.2023.102470

[B23] Kim D. Chauhan V. P. Alamani B. G. Fisher S. D. Yang Z. Jones M. R. (2025). Bio-inspired multifunctional disruptors of calcium oxalate crystallization. Nat. Commun. 16, 5229. 10.1038/s41467-025-60320-4 40473620 PMC12141576

[B24] Kozlik P. Molnarova K. Jecmen T. Krizek T. Bosakova Z. (2022). Prediction of intact N-Glycopeptide retention time windows in hydrophilic interaction liquid chromatography. Molecules 27, 3723. 10.3390/molecules27123723 35744847 PMC9228347

[B25] Li G. Wang Y. Sun C. Liu F. (2023). Determination of the microscopic acid dissociation constant of piperacillin and identification of dissociated molecular forms. Front. Chem. 11, 1177128. 10.3389/fchem.2023.1177128 37179774 PMC10169600

[B26] Li X. Qu M. Li H. Li T. (2025). Plasma pharmacokinetics of the combination of potassium chloride extended-release tablets and potassium citrate granules in patients with cardiovascular emergencies. Front. Cardiovasc Med. 12, 1636090. 10.3389/fcvm.2025.1636090 41567386 PMC12816252

[B27] Liu C. Xia Y. Hua M. Li Z. Zhang L. Li S. (2020). Functional properties and antioxidant activity of gelatine and hydrolysate from deer antler base. Food Sci. Nutr. 8, 3402–3412. 10.1002/fsn3.1621 32724604 PMC7382106

[B28] Liu H. Cao M. Jin Y. Jia B. Wang L. Dong M. (2023). Network pharmacology and experimental validation to elucidate the pharmacological mechanisms of Bushen Huashi decoction against kidney stones. Front. Endocrinol. (Lausanne) 14, 1031895. 10.3389/fendo.2023.1031895 36864834 PMC9971497

[B29] Liu Z. Du D. Zhang S. (2024). Tumor-derived exosomal miR-1247-3p promotes angiogenesis in bladder cancer by targeting FOXO1. Cancer Biol. Ther. 25, 2290033. 10.1080/15384047.2023.2290033 38073044 PMC10761019

[B30] Mahmoud El-Sayed Ali S. P. Pearson J. P. (2017). Total trypsin and active trypsin: could they be used as a biomarker for duodenal reflux. Glob. J. Otolaryngol. 3, 46–51. 10.19080/GJO.2017.03.555611

[B31] Mancini S. Schoenenberger C. E. Kolesnik-Goldmann N. Hinic V. Egli A. Nolte O. (2025). Overestimation of piperacillin/tazobactam resistance inEscherichia coliby disc diffusion and gradient strip methods. J. Antimicrob. Chemother. 80, 3464–3467. 10.1093/jac/dkaf304 40820360 PMC12670158

[B32] Maruyama M. Sawada K. P. Tanaka Y. Okada A. Momma K. Nakamura M. (2023). Quantitative analysis of calcium oxalate monohydrate and dihydrate for elucidating the formation mechanism of calcium oxalate kidney stones. PLoS One 18, e0282743. 10.1371/journal.pone.0282743 36893192 PMC9997882

[B33] Messana I. Manconi B. Cabras T. Boroumand M. Sanna M. T. Iavarone F. (2023). The post-translational modifications of human salivary peptides and proteins evidenced by top-down platforms. Int. J. Mol. Sci. 24, 12776. 10.3390/ijms241612776 37628956 PMC10454625

[B34] Moore B. W. (1965). A soluble protein characteristic of the nervous system. Biochem. Biophys. Res. Commun. 19, 739–744. 10.1016/0006-291x(65)90320-7 4953930

[B35] Negri A. L. Spivacow F. R. (2023). Kidney stone matrix proteins: role in stone formation. World J. Nephrol. 12, 21–28. 10.5527/wjn.v12.i2.21 37035509 PMC10075018

[B36] Neukam M. Sala P. Brunner A. D. Ganß K. Palladini A. Grzybek M. (2024). Purification of time-resolved insulin granules reveals proteomic and lipidomic changes during granule aging. Cell. Rep. 43, 113836. 10.1016/j.celrep.2024.113836 38421874

[B37] Pearle M. S. Goldfarb D. S. Assimos D. G. Curhan G. Denu-Ciocca C. J. Matlaga B. R. (2014). Medical management of kidney stones: AUA guideline. J. Urol. 192, 316–324. 10.1016/j.juro.2014.05.006 24857648

[B38] Phillips R. Hanchanale V. S. Myatt A. Somani B. Nabi G. Biyani C. S. (2015). Citrate salts for preventing and treating calcium containing kidney stones in adults. Cochrane Database Syst. Rev. 2015, CD010057. 10.1002/14651858.CD010057.pub2 26439475 PMC9578669

[B39] Polat S. Eral H. B. (2021). Elucidating the role of hyaluronic acid in the structure and morphology of calcium oxalate crystals. Adv. Powder Technol. 32 (10), 3650–3659. 10.1016/j.apt.2021.08.021

[B40] Ragland S. A. Criss A. K. (2017). From bacterial killing to immune modulation: recent insights into the functions of lysozyme. PLoS Pathog. 13, e1006512. 10.1371/journal.ppat.1006512 28934357 PMC5608400

[B41] Robinson T. E. Hughes E. Wiseman O. J. Stapley S. A. Cox S. C. Grover L. M. (2020). Hexametaphosphate as a potential therapy for the dissolution and prevention of kidney stones. J. Mater Chem. B 8, 5215–5224. 10.1039/d0tb00343c 32436557

[B42] Ruiz-Agudo E. Burgos-Cara A. Ruiz-Agudo C. Ibañez-Velasco A. Cölfen H. Rodriguez-Navarro C. (2017). A non-classical view on calcium oxalate precipitation and the role of citrate. Nat. Commun. 8, 768. 10.1038/s41467-017-00756-5 28974672 PMC5626694

[B43] Šálek T. Musil P. Pšenčík M. Palička V. (2022). Post-collection acidification of spot urine sample is not needed before measurement of electrolytes. Biochem. Med. (Zagreb) 32, 020702. 10.11613/BM.2022.020702 35464747 PMC8996324

[B44] Sartelli M. Catena F. Di Saverio S. Ansaloni L. Malangoni M. Moore E. E. (2014). Current concept of abdominal sepsis: WSES position paper. World J. Emerg. Surg. 9, 22. 10.1186/1749-7922-9-22 24674057 PMC3986828

[B45] Schmid-Schönbein G. W. (2016). The autodigestion hypothesis: proteolytic receptor cleavage in rheological and cardiovascular cell dysfunction1. Biorheology 53, 179–191. 10.3233/BIR-17131 28269737 PMC5389039

[B46] Shah P. Baral R. Agrawal C. S. Lamsal M. Baral D. Khanal B. (2020). Urinary calculi: a microbiological and biochemical analysis at a tertiary care hospital in Eastern Nepal. Int. J. Microbiol. 2020, 8880403. 10.1155/2020/8880403 33005194 PMC7503111

[B47] Shanthil M. Sandeep K. Sajith P. K. (2020). Cooperative effects of Na+ and citrates on the dissolution of calcium oxalate crystals. Phys. Chem. Chem. Phys. 22, 4788–4792. 10.1039/c9cp06499k 32068201

[B48] Shi X. Li X. Li X. He Z. Chen X. Song J. (2022). Antibacterial properties of TMA against *Escherichia coli* and effect of temperature and storage duration on TMA content, lysozyme activity and content in eggs. Foods 11, 527. 10.3390/foods11040527 35206004 PMC8870930

[B49] Shih H. J. Chang C. Y. Lai C. H. Huang C. J. (2021). Therapeutic effect of modulating the NLRP3-regulated transforming growth factor-β signaling pathway on interstitial cystitis/bladder pain syndrome. Biomed. Pharmacotherapy 138, 111522. 10.1016/j.biopha.2021.111522 34311526

[B50] Shpitzer S. A. Shpunt I. Loebl N. Perl L. Enikeev D. Darawsha A. E. (2025). Risk stratification for repeat stone surgery: the role of stone composition. World J. Urol. 43, 203. 10.1007/s00345-025-05573-w 40167767 PMC11961492

[B51] Siddiqui S. Ahmed N. Goswami M. Chakrabarty A. Chowdhury G. (2021). DNA damage by withanone as a potential cause of liver toxicity observed for herbal products of Withania somnifera (ashwagandha). Curr. Res. Toxicol. 2, 72–81. 10.1016/j.crtox.2021.02.002 34345852 PMC8320610

[B52] Siener R. Hesse A. (2021). Effect of black tea consumption on urinary risk factors for kidney stone formation. Nutrients 13, 4434. 10.3390/nu13124434 34959987 PMC8708000

[B53] Stephan J. R. Nolan E. M. (2016). Calcium-induced tetramerization and zinc chelation shield human calprotectin from degradation by host and bacterial extracellular proteases. Chem. Sci. 7, 1962–1975. 10.1039/C5SC03287C 26925211 PMC4763987

[B54] Sui W. Hollingsworth J. M. Oerline M. K. Hsi R. S. Crivelli J. J. Best S. L. (2025). Health care spending associated with preventative pharmacologic therapy for urolithiasis. Urol. Pract. 12, 594–602. 10.1097/UPJ.0000000000000829 40372308 PMC12353612

[B55] Tang Y. Bai Y. Feng D. Song M. Han P. (2021). Analysis of composition and related factors of 3760 cases of urinary calculi in single center. Sichuan Med. J. 42, 14–17. (in Chinese). 10.16252/j.enki.issn1004-0501-2021.01.004

[B56] Trombetta A. Benvenuto S. Barbi E. (2021). Recurrent pain in a child with cerebral palsy: answers. Pediatr. Nephrol. (Berlin, Germany) 36, 4063–4065. 10.1007/s00467-021-05156-y 34324051 PMC8599261

[B57] Tunnicliffe D. J. Mallett A. Cashmore B. Mullan A. Lloyd L. Yip A. (2024). Update thiazide diuretic evidence review for CARI guidelines kidney stones recommendations. Kidney Int. Rep. 9, 1145–1148. 10.1016/j.ekir.2024.02.1398 38707826 PMC11068971

[B58] Wang Y. Yang T. He Q. (2020). Strategies for engineering advanced nanomedicines for gas therapy of cancer. Natl. Sci. Rev. 7, 1485–1512. 10.1093/nsr/nwaa034 34691545 PMC8291122

[B59] Wang F. Zhang G. Xu T. Ma J. Wang J. Liu S. (2024). High and selective cytotoxicity of *ex vivo* expanded allogeneic human natural killer cells from peripheral blood against bladder cancer: implications for natural killer cell instillation after transurethral resection of bladder tumor. J. Exp. and Clin. Cancer Res. 43, 24. 10.1186/s13046-024-02955-7 38245792 PMC10799482

[B60] Wang Y. Zhu Y. Luo W. Long Q. Fu Y. Chen X. (2024). Analysis of components and related risk factors of urinary stones: a retrospective study of 1055 patients in southern China. Sci. Rep. 14, 28357. 10.1038/s41598-024-80147-1 39550454 PMC11569250

[B61] Wei W. Huang J. Zhong Y. Mai Y. Xu Z. (2019). The application value of CT hounsfield unit for flexible ureteroscopic lithotripsy in the treatment of renalpelvis calculi (2-3 cm). Chin. J. Endourol. 13, 95–98. 10.3877/cma.j.issn.1674-3253.2019.02.006

[B62] Wesson J. A. (2025). Comparison of calcium oxalate stone matrix proteomics data to predictions from *in vitro* studies of protein–crystal interactions. Cryst. Growth and Des. 25, 452–461. 10.1021/acs.cgd.4c01077

[B63] Wesson J. A. Zenka R. Lulich J. Eisenhauer J. Davis C. (2022). Comparison of cat and human calcium oxalate monohydrate kidney stone matrix proteomes. Urolithiasis 50, 653–664. 10.1007/s00240-022-01363-w 36180755 PMC10173728

[B64] Xia L. Xuan H. Cao Y. Du Z. Zhong H. Chen Q. (2022). Computational analysis of influencing factors and multiple scoring systems of stone clearance rate after flexible ureteroscopic lithotripsy. Comput. Intell. Neurosci. 2022, 7879819. 10.1155/2022/7879819 36199957 PMC9529465

[B65] Yang T. Yan W. (2025). Strategies for enhancing the antibacterial efficacy of lysozyme and the resulting outcome. Int. J. Biol. Macromol. 310, 143137. 10.1016/j.ijbiomac.2025.143137 40233915

[B66] Yang C. Zhao J. Lin C. Gao Y. Luo J. He F. (2023). Inhibition of integrin receptors reduces extracellular matrix levels, ameliorating benign prostate hyperplasia. Int. J. Biol. Macromol. 253, 126499. 10.1016/j.ijbiomac.2023.126499 37659484

[B67] Zhang Y. Ding H. (2016). Comparative study on the *in vitro* litholytic effects of water extracts of Phragmites communis and Polygonum cuspidatum on different types of urinary calculi. J. Pract. Med. Tech. 23, 1183–1185. (in Chinese).

[B68] Zhang J. Li K. Chen H. Hu X. Guo Z. Chen S. (2023). Retrospective analysis of urinary tract stone composition in a Chinese ethnic minority colony based on fourier transform infrared spectroscopy. Sci. Rep. 13, 13453. 10.1038/s41598-023-40603-w 37596395 PMC10439141

[B69] Zhao W. You P. (2021). 16SrRNA sequencing analysis of urinary microbiome and stone microbiome of kidney stone patients. Intemational Joumal Urology Nephrol. 41, 650–653. (in Chinese). 10.3760/cma.j.cn431460-20201203-00021

[B70] Zhao J. Chen S. Yang C. Zhou M. Yang T. Sun B. (2022). Activation of CXCL13/CXCR5 axis aggravates experimental autoimmune cystitis and interstitial cystitis/bladder pain syndrome. Biochem. Pharmacol. 200, 115047. 10.1016/j.bcp.2022.115047 35452631

[B71] Zhao J. Zhou M. Yang C. Liu Y. W. Yang T. Sun B. (2025). S100A9 as a potential novel target for experimental autoimmune cystitis and interstitial cystitis/bladder pain syndrome. Biomark. Res. 13, 72. 10.1186/s40364-025-00763-5 40346703 PMC12065242

[B72] Zhou X. J. Zhang J. Zhang C. Xu C. G. (2014). *In vitro* dissolution of calcium oxalate stones with ethylenediaminetetraacetic acid and snake venom thrombin-like enzyme. Urol. Int. 92, 349–355. 10.1159/000353096 24246673

